# Statins markedly potentiate aminopeptidase inhibitor activity against (drug-resistant) human acute myeloid leukemia cells

**DOI:** 10.20517/cdr.2023.20

**Published:** 2023-07-04

**Authors:** Gerrit Jansen, Marjon Al, Yehuda G. Assaraf, Sarah Kammerer, Johan van Meerloo, Gert J. Ossenkoppele, Jacqueline Cloos, Godefridus J. Peters

**Affiliations:** ^1^Department of Rheumatology and Clinical Immunology, Amsterdam Rheumatology and immunology Center, Amsterdam University Medical Center, location VUmc, Amsterdam 1081 HV, The Netherlands.; ^2^The Fred Wyszkowsky Cancer Research Laboratory, Faculty of Biology, The Technion-Israel Institute of Technology, Haifa 3200003, Israel.; ^3^Department of Medical Oncology, Amsterdam University Medical Center, location VUmc, Amsterdam 1081 HV, The Netherlands.; ^4^Institute of Biotechnology, Molecular Cell Biology, Brandenburg University of Technology Cottbus-Senftenberg, Senftenberg 01968, Germany.; ^5^Department of Hematology, Amsterdam University Medical Center, location VUmc, Amsterdam 1081 HV, The Netherlands.; ^6^Department of Biochemistry, Medical University of Gdansk, Gdansk 80-210, Poland.

**Keywords:** Aminopeptidase, statins, mevalonate pathway, carboxyl esterase, Rheb, mTOR

## Abstract

**Aim:** This study aimed to decipher the molecular mechanism underlying the synergistic effect of inhibitors of the mevalonate-cholesterol pathway (i.e., statins) and aminopeptidase inhibitors (APis) on APi-sensitive and -resistant acute myeloid leukemia (AML) cells.

**Methods:** U937 cells and their sublines with low and high levels of acquired resistance to (6S)-[(R)-2-((S)-Hydroxy-hydroxycarbamoyl-methoxy-methyl)-4-methyl-pentanoylamino]-3,3 dimethyl-butyric acid cyclopentyl ester (CHR2863), an APi prodrug, served as main AML cell line models. Drug combination effects were assessed with CHR2863 and *in vitro* non-toxic concentrations of various statins upon cell growth inhibition, cell cycle effects, and apoptosis induction. Mechanistic studies involved analysis of Rheb prenylation required for mTOR activation.

**Results:** A strong synergy of CHR2863 with the statins simvastatin, fluvastatin, lovastatin, and pravastatin was demonstrated in U937 cells and two CHR2863-resistant sublines. This potent synergy between simvastatin and CHR2863 was also observed with a series of other human AML cell lines (e.g., THP1, MV4-11, and KG1), but not with acute lymphocytic leukemia or multiple solid tumor cell lines. This synergistic activity was: (i) specific for APis (e.g., CHR2863 and Bestatin), rather than for other cytotoxic agents; and (ii) corroborated by enhanced induction of apoptosis and cell cycle arrest which increased the sub-G1 fraction. Consistently, statin potentiation of CHR2863 activity was abrogated by co-administration of mevalonate and/or farnesyl pyrophosphate, suggesting the involvement of protein prenylation; this was experimentally confirmed by impaired Rheb prenylation by simvastatin.

**Conclusion:** These novel findings suggest that the combined inhibitory effect of impaired Rheb prenylation and CHR2863-dependent mTOR inhibition instigates a potent synergistic inhibition of statins and APis on human AML cells.

## INTRODUCTION

Targeting of protein degradation pathways has provided new therapeutic opportunities for hematological malignancies. Proteasome inhibitors, with Bortezomib (Velcade®) as a prototypical representative, gained an established place in chemotherapeutic treatment regimens of multiple myeloma^[1,2^]. Aminopeptidases operating downstream of the proteasome were also identified as druggable targets, with Bestatin as the founding drug displaying activity mainly against solid tumors^[3,4]^. Tosedostat represents a next-generation aminopeptidase inhibitor (APi) that displays activity as monotherapy as well as in combination with various chemotherapeutic drugs, including cytarabine, daunorubicin and histone deacetylase (HDAC) inhibitors^[5-9]^. Moreover, Tosedostat demonstrated clinical activity in phase I-III combination chemotherapy for acute myeloid leukemia (AML)^[6,10-18]^ and multiple myeloma (MM)^[19]^, and has been evaluated for the treatment of solid tumors^[20,21]^. Tosedostat, and a close structural analogue (6S)-[(R)-2-((S)-Hydroxy-hydroxycarbamoyl-methoxy-methyl)-4-methyl-pentanoylamino]-3,3 dimethyl-butyric acid cyclopentyl ester (CHR2863), are APi prodrugs with an esterase-sensitive motif^[5]^. As hydrophobic drugs, they can freely diffuse into cells wherein they are converted by intracellular esterases to their hydrophilic acid active metabolites that enhance their cellular retention and promote inhibition of multiple aminopeptidases. The latter provokes an amino acid deprivation response, inhibition of mTOR activity, and blockade of protein synthesis^[5]^. Recently, we demonstrated^[22]^ that the cytotoxic activity of CHR2863 against U937 myeloid cells relies on carboxylesterase 1 (CES1) activity. Consistently, down-regulation of CES1 and loss of CHR2863 conversion to its hydrophilic active metabolite constituted a dominant mechanism of acquired resistance to CHR2863^[22]^**.** CES1 has an essential physiological function in cholesterol metabolism by converting cholesteryl esters to cholesterol^[23,24]^. Since AML cells harbor an aberrant cholesterol metabolism^[25-27]^, we explored whether or not statins as inhibitors of the mevalonate-cholesterol biosynthetic pathway potentiate the cytotoxic activity of APi (pro) drugs. In earlier studies, statins displayed differential sensitization of AML cells^[28-32]^ but were also able to enhance the sensitivity of various anti-leukemic drugs, including cytarabine, daunorubicin and the cell cycle inhibitor UCN-01^[33,34]^. Here, we discovered that various statins markedly potentiated the cytotoxic activity of CHR2863 in multiple human AML cell lines as well as in CHR2863-resistant cells, by a mechanism that involves perturbation of Rheb prenylation as an essential complementary factor to mTOR inhibition by APis. These novel findings uncover a potent therapeutic combination of statins and APis which may warrant a further clinical evaluation in AML treatment.

## METHODS

### Chemicals

Simvastatin (430-104-M) was obtained from Alexis Biochemicals (San Diego, CA USA). Fluvastatin (10010337) and lovastatin (10010338) were purchased from Cayman Chemical Co (Ann Arbor, MI, USA). Pravastatin (P4498), R-mevalonic acid (50838), squalene (S3632), farnesyl pyrophosphate (F6892), geranylgeranyl pyrophosphate (G6025), FTI-277 (F9803), bestatin (B8385) and daunorubicin (30450) were from Sigma Chemical Co (St. Louis MO, USA). Bortezomib was obtained from the VUmc Pharmacy department. CHR2863, (6S)-[(R)-2-((S)-Hydroxy-hydroxycarbamoyl-methoxy-methyl)-4-methyl-pentanoylamino]-3,3 dimethyl-butyric acid (CHR6768), and (S)-[3-(7-Hydroxycarbamoyl-heptanoylamino)-benzylamino-phenyl acetic acid cyclopentyl ester (CHR2875)^[5,35]^ were provided by Dr. A. Drummond (Chroma Pharmaceuticals Ltd, Abingdon, UK) and dissolved in dimethyl sulfoxide as 10 mM stock solutions and stored at -20 ^o^C.

### Antibodies

The following antibodies were used for Western blot analysis: CES1 monoclonal antibody (Lifespan Biosciences, Seattle, WA, USA, LS-C498701, 1:1,000 dilution) and rabbit polyclonal antibodies at 1:1000 dilutions: total Akt (#9272), phospho-Akt (Ser308) (C31E5E) (#2965), phospho-Akt (Ser473) (#9271), total mTOR (7C10) (#2983), phospho-mTOR (Ser2448) (#2971), phospho-mTOR (Ser2481) (#2974), total S6K (#9202), phospho-S6Kp70 (Th389) (#9205) and Rheb (#4935) all from Cell Signalling Technology, Danvers, MA, USA. β-Actin antibody was from Sigma-Aldrich, St. Louis, MO, USA (A2172, 1:10,000 dilutions). Secondary antibodies included goat anti-mouse or goat anti-rabbit antibodies conjugated to IRDye®800CW (1:10.000, Odyssey; LI-COR, Biosciences, Nebraska, USA).

### Cell culture

Human U937 myelomonocytic leukemia cells (ATCC, Manassas, VA, USA) and 2 sublines U937/CHR2863^R0.2^ and U937/CHR2863^R5^ (resistant to 0.2 and 5μM CHR2863, respectively and characterized by 14- and 270-fold acquired resistance to CHR2863) were isolated and cultured as described previously^[22]^. Other human myeloid leukemia cell lines (THP1, MV4-11, and KG1), human lymphoblastic cell lines (CEM and CEM/Vbl), human ovarian carcinoma cell lines (2008 and 2008/MRP1), human breast carcinoma cell lines (MCF7/WT and MCF7/MR), human lung cancer (SW1573) and human nasopharyngeal carcinoma cells (KB) were cultured as described previously^[36-41]^. Briefly, cells were grown in RPMI-1640 medium (Lonza, Verviers, Belgium) supplemented with 10% fetal calf serum (FCS, PAA Cell Culture Company, Pasching, Austria), 20 mM HEPES, 2 mM L-glutamine, and 100 U/mL penicillin/streptomycin (all from Lonza, Verviers, Belgium). The cell lines were cultured in 25 cm^2^ culture flasks (Greiner Bio-One GmbH, Frickenhansen, Germany) in 10 mL medium at an initial density of 3 × 10^5^ cells/mL (or 1.25 × 10^4^/cm^2^ for adherent cells) and in a humidified atmosphere at 37 °C and 5% CO_2_. Cell cultures were passaged every 3-4 days. Cells were regularly checked, and found negative, for mycoplasma contamination.

### Western blotting

Western blot analysis was performed essentially as described by Verbrugge et al.^[22]^. Briefly, cell lysates were prepared from 5 × 10^6^ cells suspended in 150 µL ice-cold lysis buffer (Cell Signalling Technology, #9803) containing 4% Protease Inhibitor Cocktail (PIC) and 1 mM NaVO_4_. Supernatant fractions were collected by centrifugation (13,000 × g for 10 min, 4 ^o^C), and 30 μg protein aliquots were resolved on a 4%-20% TGX pre-cast SDS PAGE gels (Bio-Rad), followed by transfer onto a polyvinylidene difluoride (PVDF) membrane (Millipore, Billerica, MA, USA) suitable for chemiluminescent detection by the Odyssey Infrared Imaging System (PerkinElmer, Zaventem, Belgium). The membranes were pre-incubated in blocking buffer (Odyssey Blocking Buffer, LI-COR, Biosciences, Nebraska, USA) for 1 hr. Next, membranes were incubated overnight (4 ^o^C) with primary antibodies and β-actin for control of equal loading. After three washing steps (PBS/0.05% Tween20), the membranes were incubated (1 hr) with secondary antibodies, followed by antibody detection with the LI-COR Odyssey scanner (Biosciences) and digital image acquisition/quantification with the Odyssey infrared imaging system software (version 3.0.16, LI-COR Biosciences) according to the manufacturer’s instructions.

### Apoptosis assay

Cells were collected and washed three times with ice-cold PBS. Early phase apoptosis was determined by the Annexin-V/7AAD Kit (PN IM3614, Beckman Coulter) using a FACSCalibur flow cytometer (Becton and Dickinson, San Jose, CA) using the manufacturer’s protocol. Briefly, cells were washed and resuspended in binding buffer. Annexin-V (1:10) and 7-Amino-Actinomycin (7AAD, 1:20) were added and incubated for 15 min on ice in the dark. Binding buffer was added and analyzed by flow cytometry followed within 1 hr. Annexin-V-positive and 7AAD-positive cells were considered as apoptotic cells.

### Cell cycle analysis

Cell cycle analysis was performed using a FACSCalibur flow cytometer and propidium Iodide (PI) staining^[42]^. Cells were washed three times with ice-cold PBS and resuspended in medium. PI (5% Propidium Iodide dissolved in PBS with 1% trisodium citrate, 0.1% RNAse and 0.1% Triton X-100) was added, and cells were vortexed and measured directly by flow cytometry. Fluorescence signal was detected through the FL2 channel. FACS analysis was performed using Cell Quest software.

### Miscellaneous assays

Quantitative RT-PCR analysis to assess CES1 mRNA levels and LC-MS/MS analyses to determine the conversion of the prodrug CHR2863 to its metabolite CHR6768 were performed essentially as described before^[22]^.

### Statistical analysis of synergism

Combination indices (CI) for analysis of synergism between simvastatin and CHR2863 were calculated by CalcuSyn software (Version 1.1.1, copyright Biosoft 1996)^[43]^ and the multiplicative model to predict the effect of drug combinations^[44]^.

### Statistics

A two-tailed paired Student’s t-test was used for comparison between groups. Significant differences were defined at *P* < 0.05.

## RESULTS

### Simvastatin synergizes CHR2863 growth inhibition in parental and CHR2863-resistant U937 cells

**Figure 1:**
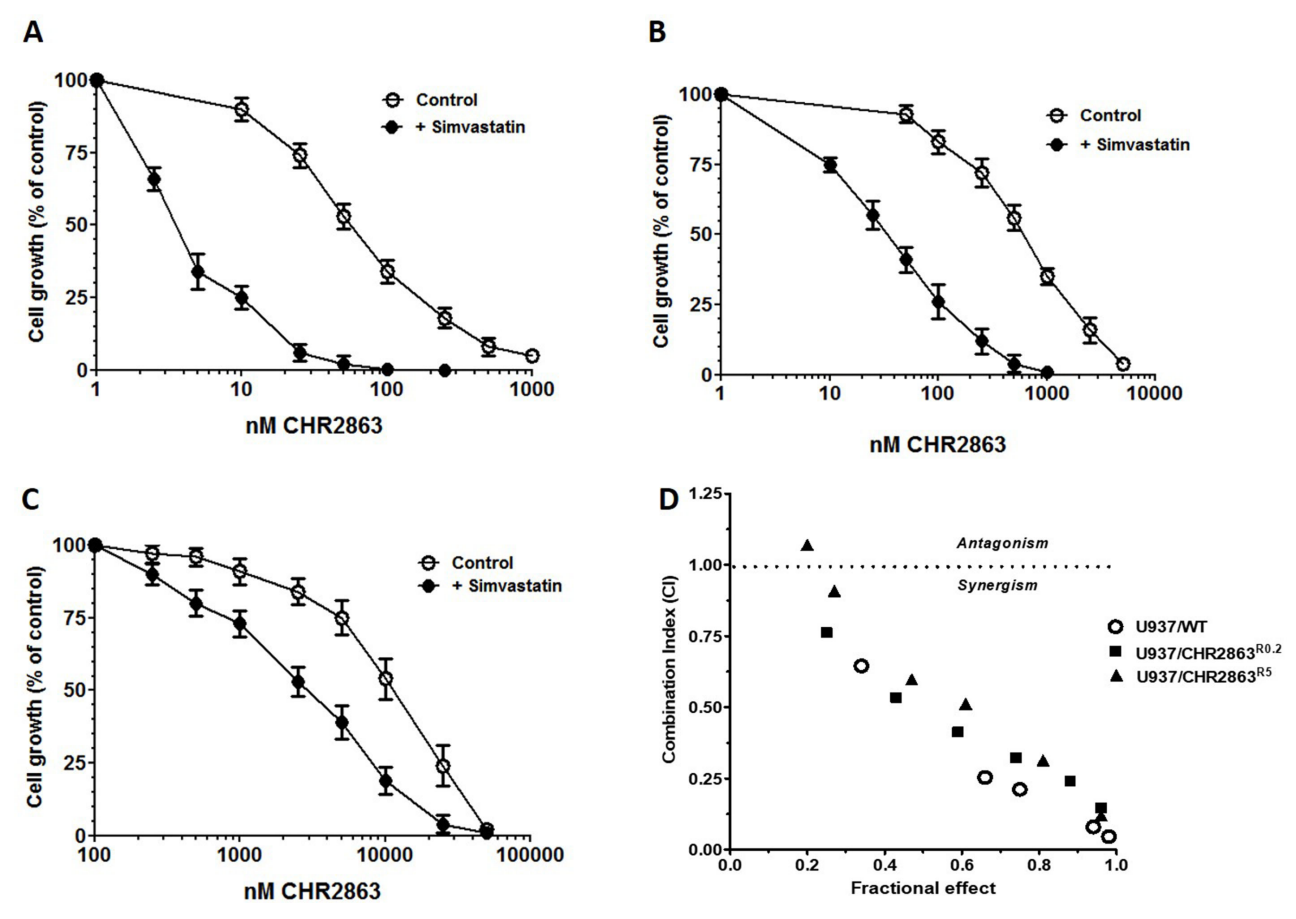
Growth inhibitory effects of CHR2863 for (A) U937/WT, (B) U937/CHR2863^R0.2^ and (C) U937/CHR2863^R5^ cells in the absence and presence of maximal non-toxic concentrations of simvastatin (2 µM, 2.5 µM, and 2.5 µM, respectively). Cell growth inhibition was determined after 72 h of drug exposure. The results depicted are the mean ± SE of 6-10 separate experiments; (D) Combination index - fraction affected plot from (A-C) of the combination simvastatin (fixed concentration) and CHR2863 for U937/WT, U937/CHR2863^R0.2^ and U937/CHR2863^R5^ cells. CHR2863: (6S)-[(R)-2-((S)-Hydroxy-hydroxycarbamoyl-methoxy-methyl)-4-methyl-pentanoylamino]-3,3 dimethyl-butyric acid cyclopentyl ester.

Growth inhibitory effects of CHR2863 were determined in human U937/WT cells and two variants, one low (U937/CHR2863^R0.2^ and one highly (U937/CHR2863^R5^) CHR2863-resistant U937 cells^[22]^ in the absence or presence of a maximal *in vitro* non-toxic concentration of 2.0-2.5 µM simvastatin [Figure 1]. For U937/WT cells [Figure 1A], simvastatin potentiated the growth inhibitory effects of CHR2863 by 14-fold (from IC_50_: 60.9 ± 15.8 nM to 4.3 ± 1.3 nM). Consistently, simvastatin potentiated CHR2863 activity 18-fold in U937/CHR2863^R0.2^ cells (from IC_50_: 682 ± 182 nM to an IC_50_ of 37.8 ± 10.8 nM), which compares to the sensitivity of U937/WT cells to CHR2863 [Figure 1B]. Lastly, simvastatin also potentiated the growth inhibitory effect of CHR2863 in U937/CHR2863^R5^ cells, albeit with a lower potentiation factor, 3.3-fold (from IC_50_: 12,900 ± 4,300 nM to 3,900 ± 2,200 nM) [Figure 1C]. Analysis of the dose-response effect of drug interactions at a constant dose of simvastatin and fractional effect by CHR2863 revealed remarkable combination indices (CI) well below 1 for parental and CHR2863-resistant U937 cells, indicating a strong synergistic interaction [Figure 1D], especially at the FA > 0.5, which is considered as relevant because growth is almost completely inhibited.

### Multiple statins synergize with CHR2863 in U937/WT and CHR2863-resistant U937 cells

**Figure 2:**
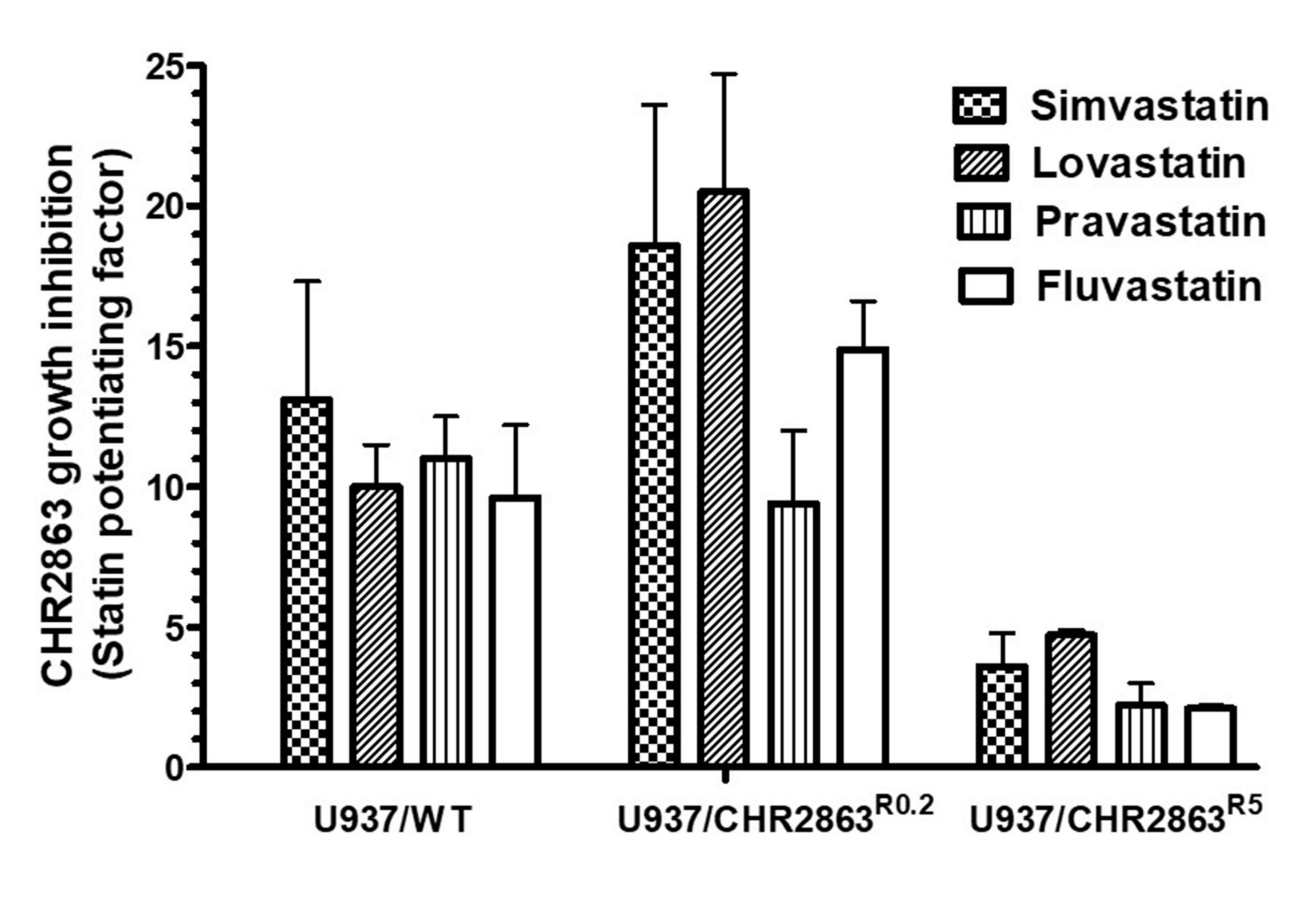
The potentiating effect of maximal non-toxic concentrations of various statins on the CHR2863 activity in U937/WT, U937/CHR2863^R0.2^ and U937/CHR2863^R5^ cells. The concentrations of simvastatin, lovastatin, pravastatin and fluvastatin were 2 µM, 2.5 µM, 100 µM and 0.5 µM, respectively, for U937/WT cells, and 2.5 µM, 5 µM, 200 µM and 1 µM, respectively, for U937/CHR2863^R0.2^ and U937/CHR2863^R5^ cells. CHR2863 dose response curves in combination with statins were generated over a CHR2863 concentration range of 0-1 μM for U937/WT cells, 0-5 μM for U937/CHR2863^R0.2^ cells and 0-50 μM CHR2863 for U937/CHR2863^R5^ cells, essentially as shown in Figure 1. Statin potentiating factor is defined as the ratio of IC_50_ (50% growth inhibition) of cell culture without statins *vs.* IC_50_ of cell cultures in the presence of statins. Cell growth inhibition was determined after 72 h of drug exposure. The results depicted are the mean ± SD of 3-4 independent experiments. CHR2863: (6S)-[(R)-2-((S)-Hydroxy-hydroxycarbamoyl-methoxy-methyl)-4-methyl-pentanoylamino]-3,3 dimethyl-butyric acid cyclopentyl ester.

We next assessed whether statins other than simvastatin also have the ability to synergize with CHR2863 activity in U937/WT and CHR2863-resistant cells. Maximal *in vitro* non-toxic concentrations of the naturally-derived statins lovastatin (2.5-5 µM) and pravastatin (100-200 µM), as well as the synthetic statin fluvastatin (0.5-1 µM) exhibited comparable capacities as simvastatin to potentiate CHR2863 activity as revealed by their potentiation factors (ratio IC_50_ CHR2863 with statin over IC_50_ CHR2863 without statin) [Figure 2].

### Statin potentiation is selective for aminopeptidase inhibitors

**Figure 3:**
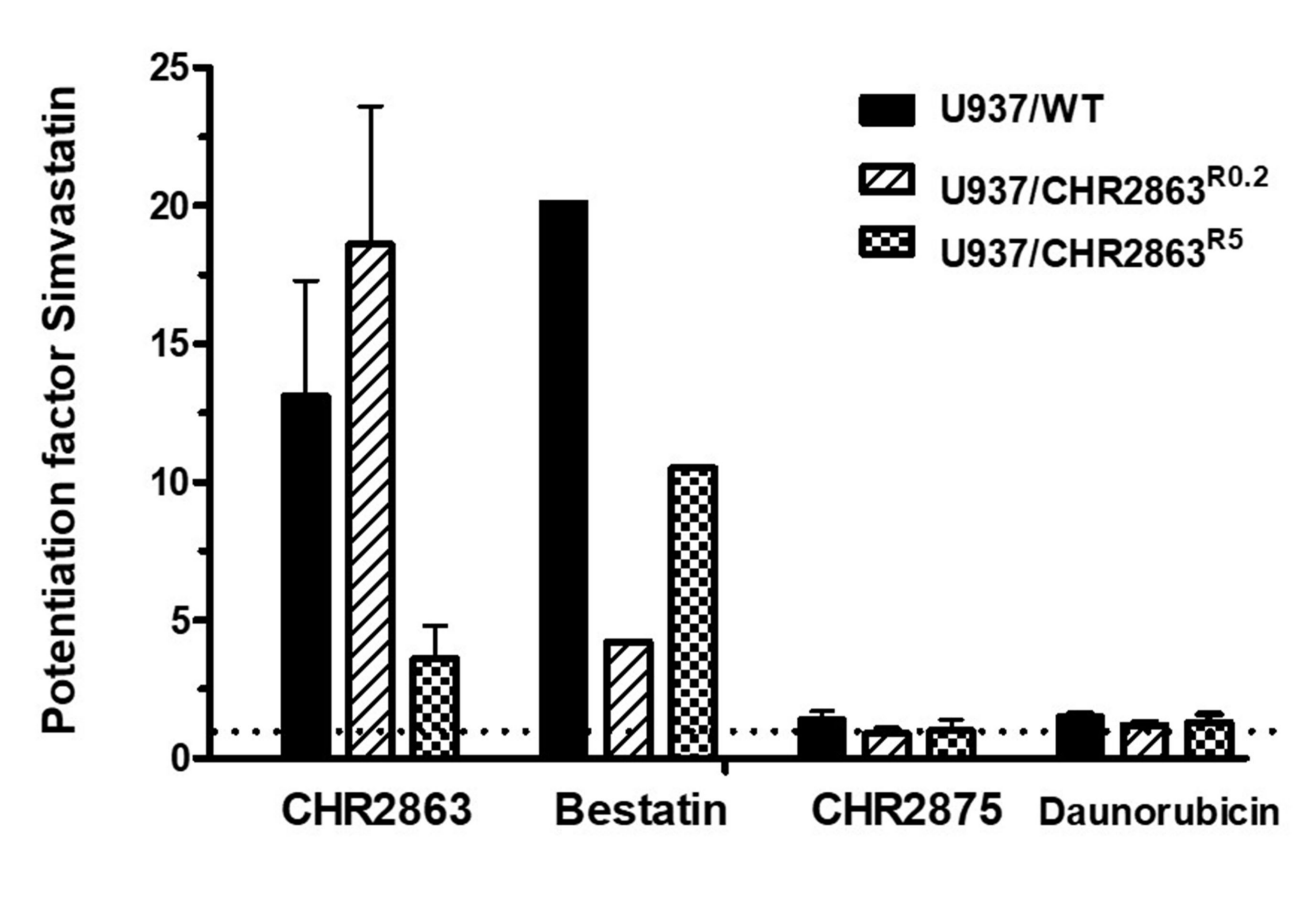
Selectivity of simvastatin-potentiating effect for APis. Effect of non-toxic concentrations of simvastatin (2-2.5 µM) on the growth inhibitory activity of the APis CHR2863 and bestatin, HDAC inhibitor prodrug CHR2875, and daunorubicin in U937/WT, U937/CHR2863^R0.2^ and U937/CHR2863^R5^ cells. Simvastatin potentiation factor is defined as the ratio of IC_50_ (50% growth inhibition) of cell culture without statins *vs.* IC_50_ of cell cultures in the presence of statins. Cell growth inhibition was determined after 72 h of drug exposure. Results depicted are the mean of two separate experiments (for bestatin) and the mean ± SD of 3-4 separate experiments for CHR2863, CHR2875 and daunorubicin. IC50 values of U937/WT, U937/CHR2863R0.2 and U937/CHR2863R5 cells for CHR2863 are: 52 ± 16 nM, 713 ± 212 nM, and 14,047 ± 5,521 nM, respectively; for Bestatin: 158 ± 15 μM, 169 ± 32 μM, and 177 ± 14 μM, respectively; for CHR2875: 158 ± 9 nM, 86 ± 13 nM, and 147 ± 36 nM, respectively; and for daunorubicin: 16 ± 1 nM, 16 ± 2 nM, and 15 ± 3 nM, respectively. APis: aminopeptidase inhibitors; CHR2863: (6S)-[(R)-2-((S)-Hydroxy-hydroxycarbamoyl-methoxy-methyl)-4-methyl-pentanoylamino]-3,3 dimethyl-butyric acid cyclopentyl ester.

To assess whether statin potentiation of the APi prodrug CHR2863 also occurs with a direct APi, we tested whether the growth inhibition by bestatin^[3,45]^ is potentiated by simvastatin. Indeed, simvastatin potentiated both CHR2863 and bestatin activities with similar potentiation factors in U937/WT and CHR2863-resistant U937 cells [Figure 3 and Supplementary Figure 1]. Moreover, statin potentiation appeared selective for APis as no potentiation was observed for two types of other drugs: CHR2875, an HDAC inhibitor prodrug^[35]^, which is bioactivated similarly as CHR2863, and daunorubicin evaluated in combination chemotherapy with Tosedostat for AML [Figure 3 and Supplementary Figure 1].

### Statin potentiation of CHR2863 activity is primarily restricted to AML cells

**Figure 4:**
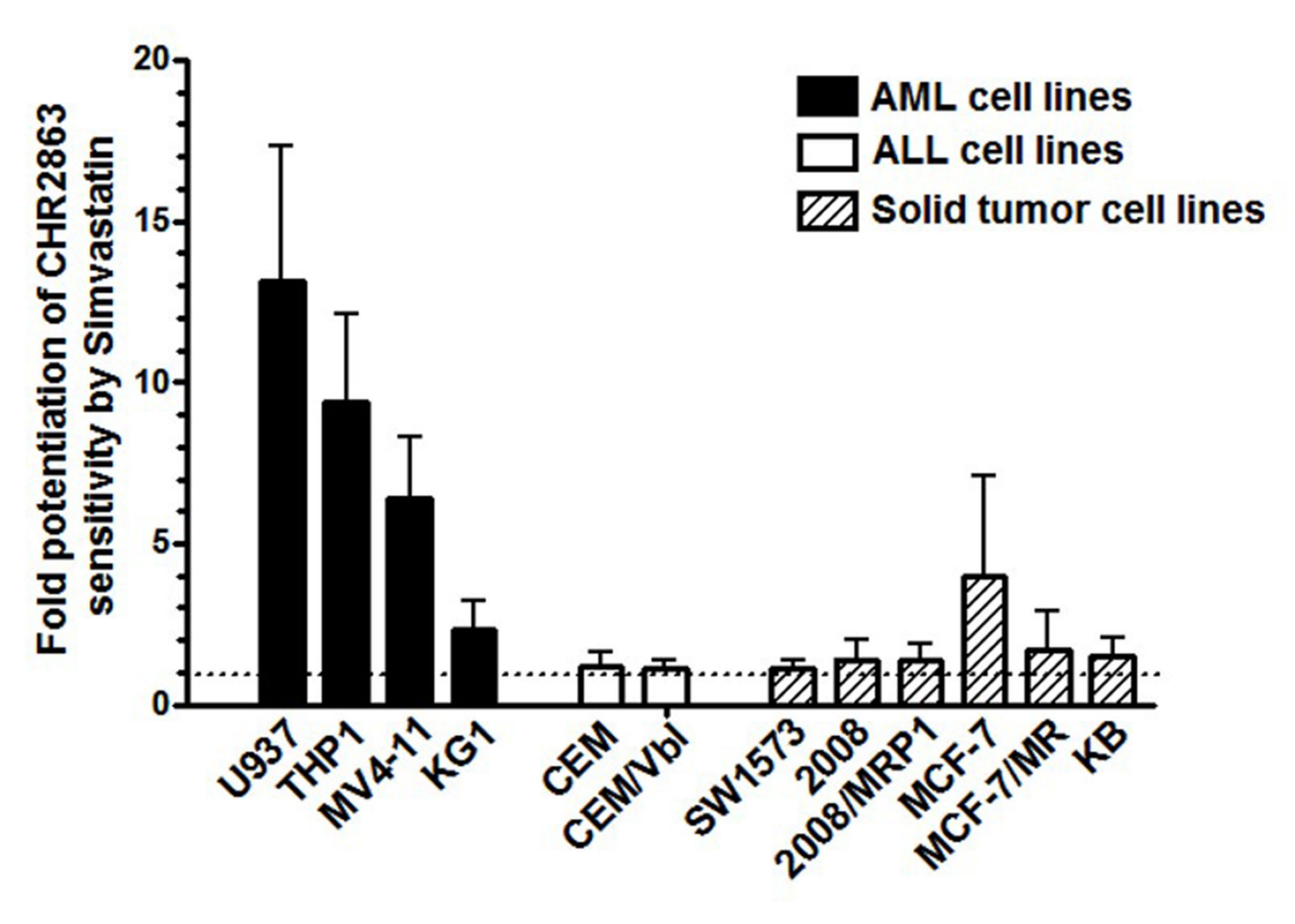
Simvastatin potentiation of CHR2863 activity in human AML cell lines *vs.* human lymphoid and solid tumor cell lines. Cell growth inhibition was determined after 72 h of drug exposure in the absence or presence of maximal non-toxic concentrations of simvastatin, being (between brackets) for: U937 (2 µM), THP1 (2.5 µM), MV4-11 (2.5 µM), KG1 (10 µM), CCRF-CEM (2.5 µM), CEM/Vbl (2.5 µM), SW1573 (0.2 µM), 2008 (0.75 µM), 2008/MRP1 (2.5 µM), MCF7 (1 µM), MCF7/MR (2.5 µM) and KB (1 µM). Simvastatin potentiation factor is defined as the ratio of IC_50_ (50% growth inhibition) of cell culture without statins *vs.* IC_50_ of cell cultures in the presence of statins. IC_50_ values (between brackets) for CHR2863 for the various cell lines (in the absence of simvastatin) were: U937 (61 ± 16 nM), THP1 (1172 ± 807 nM), MV4-11 (282 ± 51 nM), KG1 (394 ± 144 nM), CCRF-CEM (11,170 ± 5,100 nM), CEM/Vbl (29,100 ± 5,900 nM), SW1573 (6,625 ± 3,020 nM), 2008 (2,020 ± 1,080 nM), 2008/MRP1 (6,700 ± 2,560 nM), MCF7 (453 ± 400 nM), MCF7/MR (386 ± 64 nM), and KB (132 ± 50 nM). The results depicted are the mean ± SD of 3-5 independent experiments. CHR2863: (6S)-[(R)-2-((S)-Hydroxy-hydroxycarbamoyl-methoxy-methyl)-4-methyl-pentanoylamino]-3,3 dimethyl-butyric acid cyclopentyl ester.

To determine whether statin potentiation of CHR2863 activity occurs in various human AML cells other than U937 cells, the potentiating effect of maximal non-toxic concentrations of simvastatin was examined in multiple AML cell lines, acute lymphocytic leukemia (ALL) CCRF-CEM cells as well as a panel of (multidrug resistance-related) solid tumor cell lines [Figure 4 and Supplementary Figure 2]. As with U937 cells, CHR2863 growth inhibition was significantly potentiated by simvastatin in various AML cell lines, including THP1, MV4-11 and, to a lower extent, KG1 cells. In contrast, simvastatin had no potentiating effect in CCRF-CEM cells and a P-glycoprotein/MDR1-overexpressing subline CEM/VBL, although it should be emphasized that these cells had a low intrinsic sensitivity to CHR2863 (IC_50_ > 10 µM). The panel of solid tumor cell lines displayed variable sensitivity to CHR2863 (IC_50_: 0.13-6.7 µM); with the exception of MCF7/MR cells, none showed a potentiating effect by simvastatin. These results indicate that the simvastatin potentiating effect of CHR2863 is largely restricted to AML cells.

### Simvastatin - CHR2863 combinations: impact on cell growth, apoptosis and cell cycle

**Figure 5:**
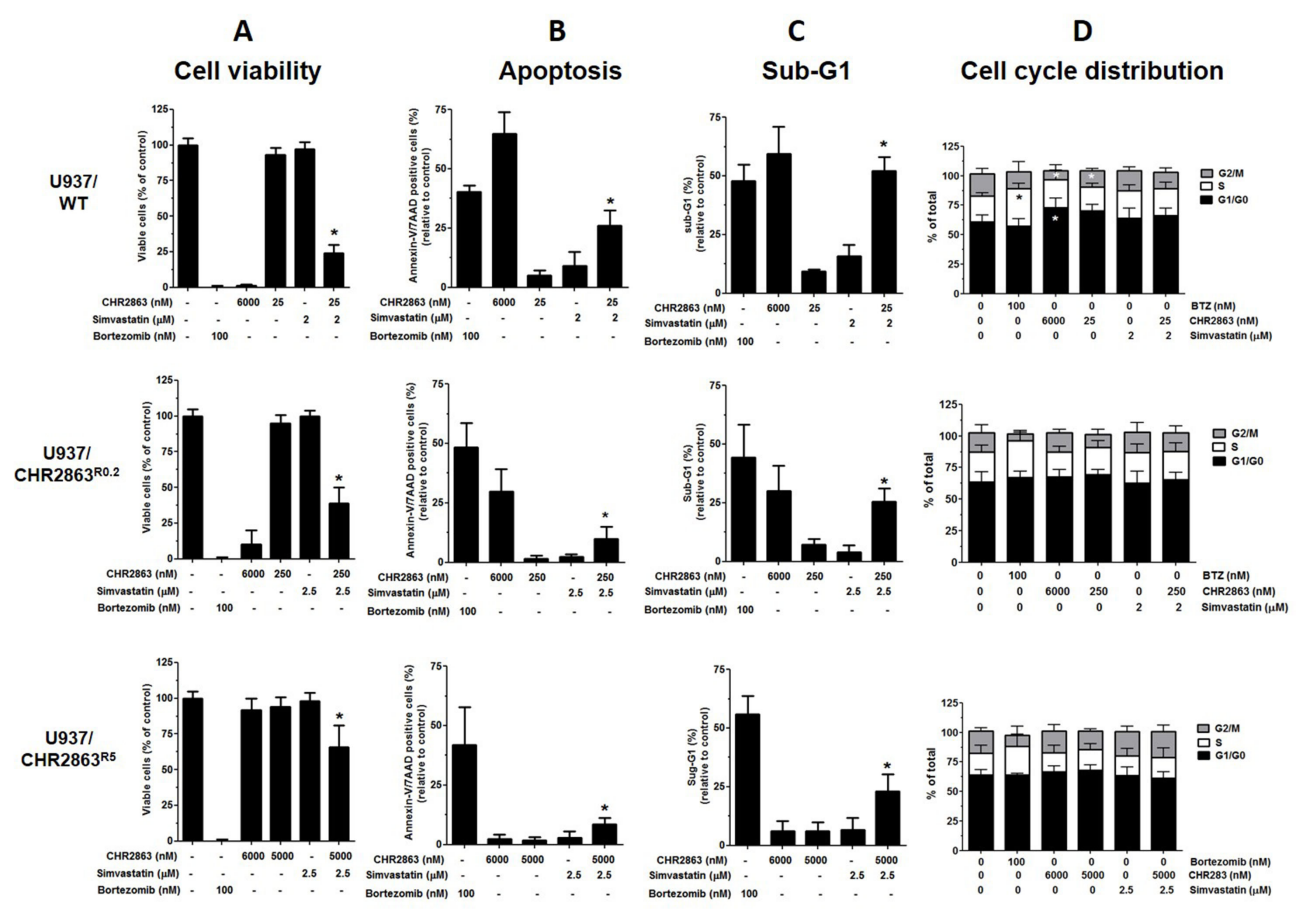
Effect of simvastatin and CHR2863 combinations on cell viability, apoptosis induction and cell cycle distribution in U937/WT, U937/CHR2863^R0.2^ and U937/CHR2863^R5^ cells. Simvastatin concentrations used for U937/WT cells, U937/CHR2863R0.2 and U937/CHR2863R5 cells were maximal in vitro non-toxic concentrations: 2 µM, 2.5 µM and 2.5 µM, respectively. For CHR2863, minimally cytotoxic (≈ IC10) were selected (from Figure 1), i.e., 25 nM, 250 nM and 5 μM for U937/WT cells, U937/CHR2863^R0.2^ and U937/CHR2863^R5^ cells, respectively. Cells (3 × 10^5^/mL in 10 mL medium) were incubated for 48 h with the indicated concentrations of simvastatin, CHR2863 and their combination and assessed for the impact on (A) cell viability, (B) apoptosis induction, (C) sub-G1 fraction and (D) cell cycle distribution. Cells incubated for 24 h with bortezomib or 48 h with 6 µM CHR2863 served as a control for cell growth inhibition and apoptosis induction. Percentages of apoptotic cells in control untreated U937/WT, U937/CHR2863^R0.2^ and U937/CHR2863^R5^ cells were 4.6% ± 1.9%, 5.2% ± 1.2% and 6.1% ± 0.9%, respectively. Sub-G1 fractions in control untreated U937/WT, U937/CHR2863^R0.2^ and U937/CHR2863^R5^ cells were 4.2% ± 2.0%, 9.1% ± 6.5% and 8.2% ± 2.2%, respectively. The results depicted are the mean ± SD of 4-5 independent experiments. *Combination statistically significant (*P* < 0.05) different compared to single drugs control cells. CHR2863: (6S)-[(R)-2-((S)-Hydroxy-hydroxycarbamoyl-methoxy-methyl)-4-methyl-pentanoylamino]-3,3 dimethyl-butyric acid cyclopentyl ester.

An exposure of 48 h to maximal *in vitro* non-toxic concentrations of simvastatin and minimally cytotoxic (≈ IC_10_) concentrations of CHR2863 was tested for the impact on cell viability, apoptosis induction and sub-G_1_ fraction/cell cycle distribution of U937/WT, U937/CHR2863^R0.2^ and U937/CHR2863^R5^ cells [Figure 5]. Bortezomib (0.1 µM) and a high concentration of CHR2863 (6 µM) were included as a reference control. Single doses of CHR2863 and simvastatin had no effect on cell viability, whereas their combination significantly reduced cell viability in all three cell lines [Figure 5A], which was accompanied by a significantly increased apoptosis [Figure 5B and Supplementary Figure 3A] and an increase in the sub-G_1_ fraction [Figure 5C and Supplementary Figure 3B]. No visible alterations in cell cycle distribution were noted at the tested concentrations of CHR2863, simvastatin or their combination [Figure 5D]. The impact of simvastatin and CHR2863 combinations on cell viability and apoptosis for the U937 cell lines were also found for three other AML cell lines; THP1 and MV4-11 and to a lesser extent for KG1 cells [Supplementary Figure 3C].

### Reversal of simvastatin potentiation of CHR2863 activity by mevalonic acid, farnesyl pyrophosphate and geranylgeranyl pyrophosphate

**Figure 6:**
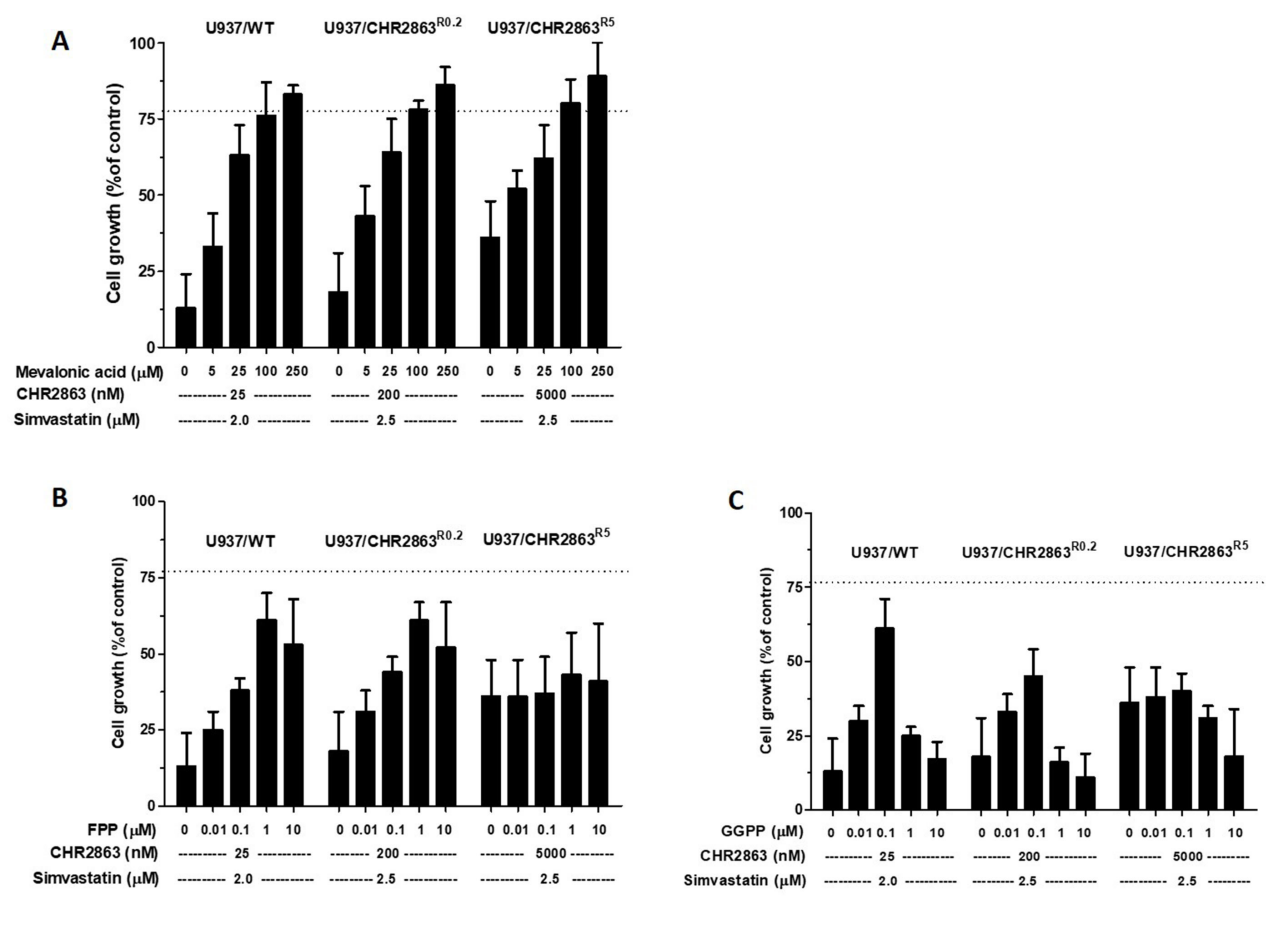
Effects of mevalonic acid, farnesyl pyrophosphate and geranylgeranyl pyrophosphate on simvastatin potentiation of CHR2863 activity. U937/WT, U937/CHR2863^R0.2^ and U937/CHR2863^R5^ cells were incubated for 72 h with the indicated concentrations of CHR2863 and non-toxic concentration of simvastatin in the presence of increasing concentrations of (A) mevalonic acid, (B) farnesyl pyrophosphate (FPP) and (C) geranylgeranyl pyrophosphate (GGPP). Results, presented as cell growth relative to control, are the mean ± SD of 4 independent experiments. The dashed line indicates the mean growth inhibition by CHR2863 alone. CHR2863: (6S)-[(R)-2-((S)-Hydroxy-hydroxycarbamoyl-methoxy-methyl)-4-methyl-pentanoylamino]-3,3 dimethyl-butyric acid cyclopentyl ester.

To determine whether or not the statin-induced inhibition of HMG-CoA reductase is implicated in the simvastatin potentiation of CHR2863 cytotoxicity, we assessed whether or not intermediates of the mevalonate pathway, i.e., mevalonic acid (MVA), farnesyl pyrophosphate (FPP) and geranylgeranyl pyrophosphate (GGPP) were able to abrogate the potentiating effect of simvastatin. Increasing concentrations of MVA fully abrogated simvastatin potentiation of CHR2863 growth inhibition in U937/WT, U937/CHR2863^R0.2^ and U937/CHR2863^R5^ cells [Figure 6A]. Likewise, increasing concentrations of FPP also abrogated the simvastatin potentiation effect of CHR2863 in U937/WT and U937/CHR2863^R0.2^ cells, albeit to a slightly lower extent than MVA [Figure 6B]. Of note, FPP failed to abrogate the simvastatin potentiation effect of CHR2863 in U937/CHR2863^R5^ cells [Figure 6B]. Lastly, GGPP abrogated the simvastatin potentiation effect of CHR2863 in U937/WT and U937/CHR2863^R0.2^ cells at an optimal concentration of 0.1 µM; above this concentration, the abrogating effect was lost [Figure 6C]. GGPP was also unable to abrogate the potentiation effect of simvastatin in U937/CHR2863^R5^ cells [Figure 6C].

Given the variable effects of FPP in abrogating the potentiating effect of simvastatin of CHR2863 in U937/WT and U937/CHR2863^R0.2^ cells *vs*. U937/CHR2863^R5^ cells, we further examined whether a farnesyltransferase inhibitor (FTI-277) had similar effects on CHR2863 potentiation in these cells. Combinations of FTI-277 and CHR2863 were synergistic in U937/WT and additive in U937/CHR2863^R0.2^ cells, whereas U937/CHR2863^R5^ cells were resistant to FTI-277 and no potentiation was found [Supplementary Figure 4].

### Simvastatin potentiation of CHR2863: mechanistic studies

To explore the mechanistic basis underlying the potentiation of CHR2863 growth inhibition by simvastatin, we first examined whether simvastatin upregulated the expression of carboxylesterase 1 (CES1), the enzyme mediating the conversion of CHR2863 to its active metabolite CHR6768^[22]^. Western blot analysis revealed that CES1 expression (as well as its other family members CES2 and CES3) in U937/WT, U937/CHR2863^R0.2^, and U937/CHR2863^R5^ cells was not altered by simvastatin and CHR2863 alone, in combination, and in combination with MVA [Supplementary Figure 5]. Notably, U937/CHR2863^R5^ cells displayed markedly decreased CES1 expression levels as shown earlier^[22]^. Consistent with unaltered CES1 expression levels in the presence of simvastatin, the ability of U937/WT and U937/CHR2863^R0.2^ cells to enzymatically convert CHR2863 to its active metabolite CHR6768 was unchanged, while U937/CHR2863^R5^ cells had lower levels in line with their lack of CES1 activity [Supplementary Figure 6].

Aminopeptidase inhibition can regulate mTOR activity^[5]^. Therefore, we evaluated whether concentrations of simvastatin and CHR2863 which showed an enhanced growth inhibitory effect (after 48 hr drug incubation) were also associated with an altered cellular phosphorylation status of intermediates of the ERK/Akt/mTOR pathway. Analysis of pERK(Thr202/Tyr204), pAkt(Ser473), pmTOR(Ser2448), pmTOR(Ser2481) and pS6Kp70(Th389) levels in U937/WT, U937/CHR2863(200), and U937/CHR2863(5µM) cells showed no major differences upon exposure to CHR2863, simvastatin, MVA, and their combinations [Supplementary Figure 7]. This suggests that other mechanisms play a prominent role in the synergy between statins and CHR2863.

**Figure 7:**
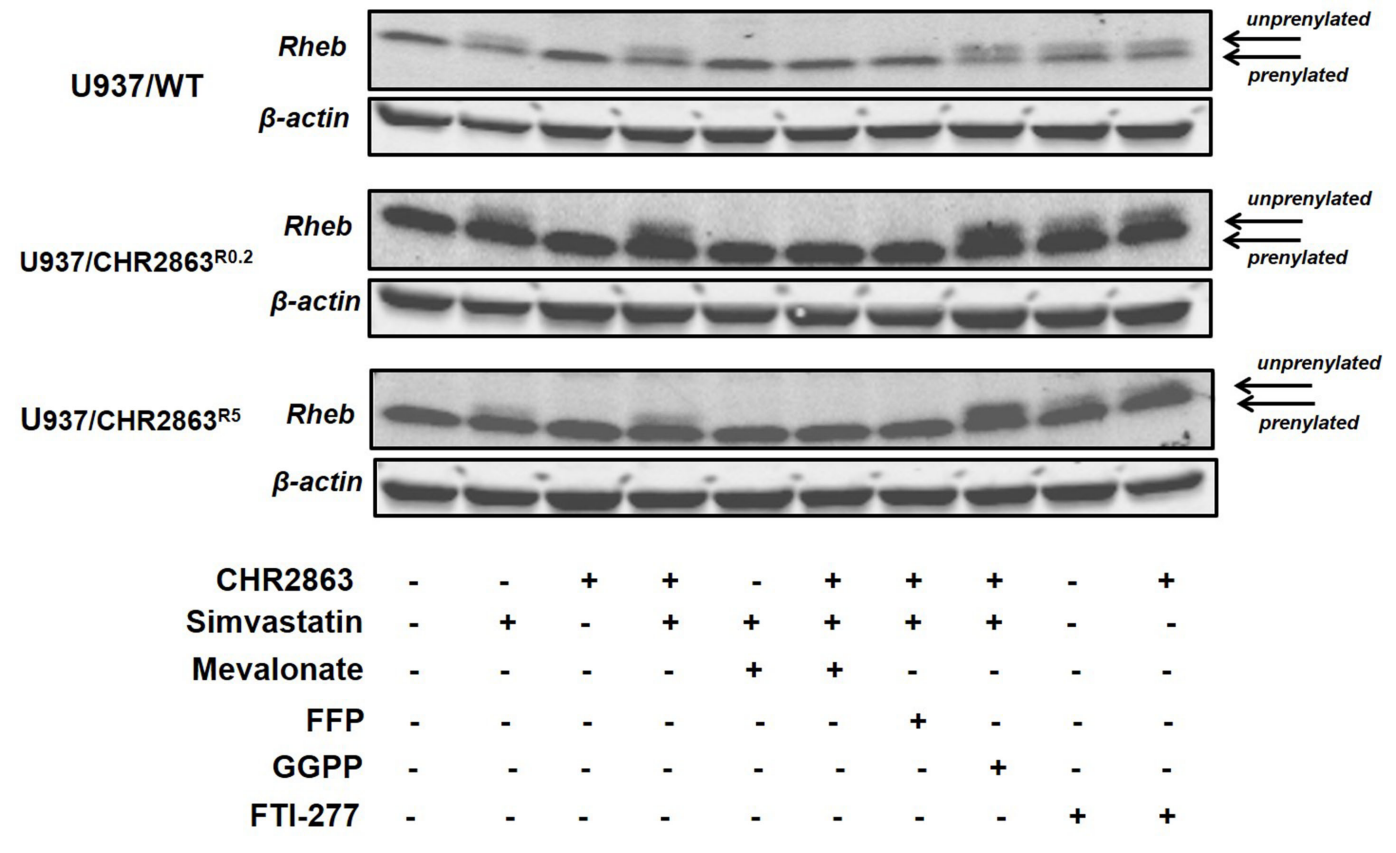
Effect of simvastatin and CHR2863 combinations on Rheb prenylation. U937/WT, U937/CHR2863^R0.2^ and U937/CHR2863^R5^ cells were incubated for 48 h with simvastatin, CHR2863, and their combination (as described in Figure 5), with or without the addition of MVA (100 µM), FPP (2 µM), GGPP (1 µM) or FTI-277 (10 µM). The slower (upper) migrating band represents unprenylated Rheb, and the faster (lower) migrating band represents prenylated Rheb. CHR2863: (6S)-[(R)-2-((S)-Hydroxy-hydroxycarbamoyl-methoxy-methyl)-4-methyl-pentanoylamino]-3,3 dimethyl-butyric acid cyclopentyl ester.

Statins are known to impair the prenylation and thus membrane localization of various proteins^[46-48]^. In the context of mTOR activation, it has been demonstrated that lysosomal membrane integration of Rheb protein is of relevance and is prenylation-dependent^[49-51]^. To this end, we examined whether under conditions that potentiated CHR2863 activity, simvastatin interfered with Rheb prenylation in U937/WT, U937/CHR2863^R0.2^ and U937/CHR2863^R5^ cells. Indeed, exposure to simvastatin resulted in a marked increase in unprenylated Rheb in all three tumor cell lines [Figure 7], as did the exposure to FTI-277. The level of unprenylated Rheb was maintained in CHR2863 + simvastatin combinations, whereas exposure to CHR2863 alone had no effect on Rheb prenylation status. MVA and FPP, but not GGPP, abrogated the unprenylation impact of simvastatin alone and in combination with CHR2863. Hence, this profile of Rheb unprenylation parallels the statin and inhibitor FTI-277-induced potentiation of CHR2863 activity in U937/WT, U937/CHR2863^R0.2^ and U937/CHR2863^R5^ cells.

**Figure 8:**
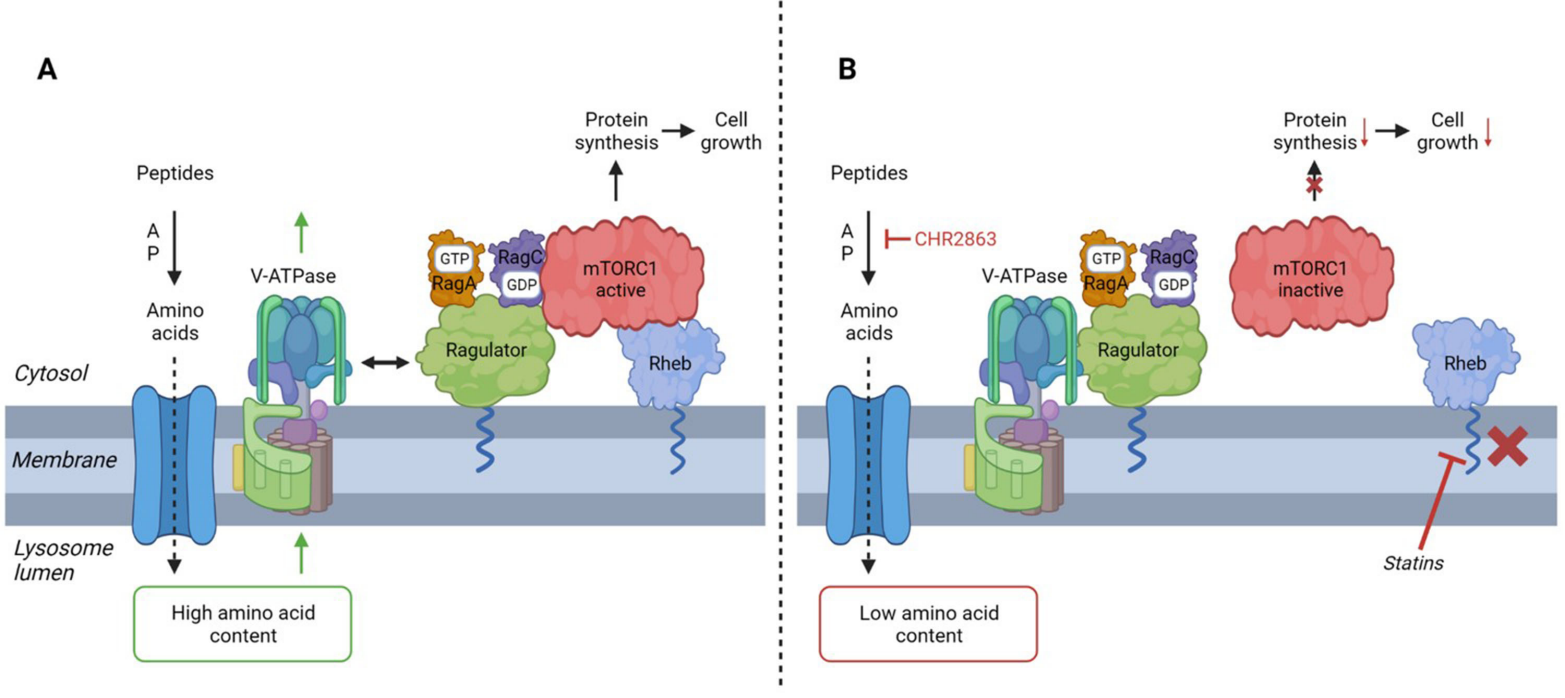
Proposed model for synergistic action of statins and APi CHR2863. (A) Peptide breakdown by aminopeptidases provides amino acids for re-utilization in protein synthesis. According to previously described models^[49-51,56,62,63,66]^, an increased intralysomal amino acid content triggers dissociation of V-ATPase and Ragulator-Rag-mTORC1 complex. Binding of the latter complex to (prenylated) Rheb (in the lysosomal membrane) and membrane association of Ragulator will then induce mTOR activation and initiation of protein synthesis; (B) Inhibition of aminopeptidases by CHR2863 (or bestatin) will reduce the intralysomal amino acid content and dissociation of the Ragulator-Rag complex from mTORC1. By a different mechanism, statins may block Rheb prenylation and abolish its lysosomal membrane localization. The combined effect of CHR2863 and statins may then synergize in impairing mTOR activation, protein synthesis and inhibiting cell growth. The figure was created via *BioRender*. APi: Aminopeptidase inhibitor; CHR2863: (6S)-[(R)-2-((S)-Hydroxy-hydroxycarbamoyl-methoxy-methyl)-4-methyl-pentanoylamino]-3,3 dimethyl-butyric acid cyclopentyl ester.

A composite summary model which proposes a mechanistic basis for the synergistic action of APis and statins in AML cells is presented and discussed in [Figure 8].

## DISCUSSION

Aberrant cholesterol metabolism is a characteristic feature of AML cells and has been exploited for therapeutic interventions with statins as inhibitors of HMG-CoA reductase, the key enzyme in the MVA-cholesterol pathway^[26,46,52]^. Both *in vitro* and *in vivo* studies demonstrated that high concentrations of statins can induce apoptosis in AML cells through perturbations of prenylation and membrane anchoring of proteins involved in signal transduction pathways. These include disruption of Ras family members and pro-survival pathways such as MEK/ERK and PI3K/Akt/mTOR^[29-31,53-56]^. Furthermore, statins also elicit additive/synergistic effects with various other chemotherapeutic drugs^[33,34,57]^. The current study is the first report to reveal that non-toxic concentrations of statins markedly potentiate the growth inhibitory effects of either a prodrug (CHR2863) or a direct inhibitor (bestatin) of aminopeptidases in human AML cells. Hence, these findings bear important implications for future therapeutics as well as overcoming chemoresistance in AML.

APis such as Tosedostat and its close structural analogue CHR2863 are prodrugs that rely on esterase activities for their conversion to active metabolites that can inhibit multiple aminopeptidases, thereby provoking amino acid depletion^[5]^. Earlier, we demonstrated that CES1 is the most likely candidate enzyme for the bio-activation of these prodrugs, given the high CES1 expression in myeloid cell lines and M4 and M5 FAB subtypes of AML clinical specimens^[22]^. The role of CES1 in this enzymatic bio-activation was further substantiated by the fact that acquired resistance to CHR2863 in AML cells was mediated by downregulation of CES1 expression. Regarding CHR2863 resistance, combinations of *in vitro* non-toxic concentrations of statins were able to sensitize 14-fold resistant U937/CHR2863^R0.2^ cells, hence restoring WT sensitivity. Highly (270-fold) CHR2863 resistant U937/CHR2863^R5^ cells could also be sensitized by co-administration of statins, albeit to a lower extent (3-4 fold), even given the fact that active metabolite formation was almost 100-fold lower than in WT cells. Statin-dependent sensitization of CHR2863-resistant cells did not involve increased CES1 expression and/or enhanced active metabolite formation, suggesting that other mechanisms account for this potentiation effect. In drug combination experiments, the combination of simvastatin and CHR2863 led to a significant enhancement of apoptosis induction as reflected in the high accumulation of cells in sub-G_1_ fraction, whereas treatment with either drug alone had a minimal effect. Beyond apoptosis, it cannot be ruled out that alternative mechanisms, e.g., ferroptosis^[58,59]^, contribute to cell death. Furthermore, cell cycle analysis of simvastatin + CHR2863 combinations did not reveal any distinct cell cycle arrest in G_1_/G_0_-, S- or G2/M-phase, suggesting that the drug treatment did not interfere with specific cell cycle phases or checkpoints.

Further experiments with intermediates of the MVA-cholesterol pathway, including FPP, GGPP and MVA, were performed to identify the mechanism underlying statin-dependent potentiation of CHR2863 activity. Apoptosis induced by statins in AML cells was reversed by the addition of MVA and GGPP rather than FFP^[28]^. With respect to simvastatin potentiation of CHR2863 activity in U937 cells, GGPP reversed the potentiation effect in a narrow concentration range around 100 nM; above this concentration, the reversal effect was lost. The mechanistic reason for this decline in reversal effect beyond 100 nM GGPP is unclear and warrants further studies. The full reversal was observed with increasing concentrations of FPP and MVA, suggesting that perturbations in protein farnesylation are involved in the potentiation effect. Interestingly though, highly CHR2863-resistant U937/CHR2863^R5^ cells were unresponsive to FPP, which could be consistent with their refractoriness to the farnesyltransferase inhibitor FTI-277 [Supplementary Figure 4]. In parental U937/WT cells, synergistic growth inhibitory effects of FTI-277 and CHR2863 combinations mimicked the simvastatin-CHR2863 combinations; however, upon the acquisition of CHR2863 resistance, the potentiation effect of FTI-277 on CHR2863 activity was gradually lost. Ding *et al*. showed that acquired resistance of U937 cells to another FTI, tipifarnib, involved alterations in Rheb prenylation and loss of inhibition of Rheb-induced mTOR signaling^[60]^. Consistently, the acquisition of CHR2863 resistance was also shown to be accompanied by activation of the Akt/mTOR pro-survival pathway, as reflected by a marked gain of sensitivity to the mTOR inhibitor rapamycin^[22]^. Given that Rheb prenylation is required for mTOR activation^[56,61-66]^, loss of prenylation through the action of statins and FTIs is likely to constitute a mechanistic basis for the synergistic effect with CHR2863, hence causing mTOR inhibition via amino acid depletion. Indeed, the present study showed [Figure 7] that the loss of Rheb prenylation provoked by simvastatin and FTI-277 was consistent with their potentiation effect on CHR2863 activity, whereas retention of Rheb prenylation by MVA or FFP abrogated this potentiating effect. However, it is remarkable that synergistic concentrations of simvastatin and CHR2863 had no apparent impact on Akt and mTOR phosphorylation patterns in U937 cells and CHR2863-resistant sublines. Therefore, further studies are required to identify other markers downstream of mTOR and to identify the mechanism of induction of apoptosis contributing to the synergy between statins and APis in AML cell lines and clinical specimens.

Many therapeutic interventions for AML are designed based on aberrant PI3K-Akt-mTOR signaling in AML cells^[67]^. Both statins and APis harbor properties interfering with this master regulator pathway and the current study provides a mechanistic rationale for their combination [Figure 8]. Whereas AML cells have shown heterogeneity in statin-induced apoptosis^[29]^, the current study indicates that non-toxic concentrations of various statins synergize with APis in multiple AML cell lines. Non-toxic concentrations of statins, as employed in the *in vitro* studies, are readily achievable *in vivo*^[26,68,69]^. One earlier clinical study showed that Tosedostat combined with cytarabine or decitabine in untreated elderly AML or high-risk MDS patients was tolerated^[14]^; however, more recent studies revealed no survival benefit^[16]^ or even inferior outcome in this patient category^[18]^. Although statin use was not reported in these studies, one could speculate that statin use, along with a high Tosedostat dosing, could contribute to over-potentiation of the drug. Therefore, it would be of interest to design a clinical study with lower doses of Tosedostat in a patient group of well-documented statin users to achieve an optimal potentiating effect and clinical benefit. Collectively, exploring the optimal combined efficacy of statins with APis in general and Tosedostat in particular deserves further exploration in the clinical setting of AML treatment.

In conclusion, this study revealed that non-toxic doses of statins could markedly potentiate the activity of aminopeptidase inhibitor (APi) drugs; both direct inhibitors like Bestatin and APi prodrugs like CHR2863 to (drug-resistant) human acute myeloid leukemia (AML) cells. The molecular basis underlying the potent synergistic inhibition of statins and APis on AML cells involved a dual inhibitory effect of impaired Rheb prenylation abrogating mTOR activation and APi-dependent mTOR inhibition. Given the fact that many cancer patients take statin medication for the treatment of other comorbidities, these novel findings call for awareness of the synergistic drug action of statins with APi-containing chemotherapeutic regimens and/or potential toxicities. These notions may warrant further evaluation in clinical studies including APis.

## DECLARATION

### Acknowledgments

Dr. Krige D (Chroma Pharmaceuticals) is acknowledged for the gift of CHR2863 and helpful discussions. Dr. Verbrugge SE, Dr. Honeywell R and Lin M are acknowledged for excellent technical assistance. Assaraf YG is the recipient of a Visiting Professor Award from the Royal Netherlands Academy of Arts and Sciences, Netherlands Organization for Scientific Research and Cancer Center Amsterdam/VU Institute for Cancer and Immunology.

### Authors’ contributions

Conception and design: Jansen G, Ossenkoppele GJ, Cloos J, Peters GJ

Funding acquisition: Jansen G, Ossenkoppele GJ, Peters GJ

Data acquisition, analysis and interpretation: Jansen G, Al M, Kammerer S, van Meerloo J, Peters GJ

Writing original draft: Jansen G, Assaraf YG, Kammerer S, Cloos J, Peters GJ

Writing, review and editing: Jansen G, Assaraf YG, Kammerer S, Ossenkoppele GJ, Cloos J, Peters GJ

Supervision: Jansen G, Assaraf YG, Cloos J, Peters GJ

Visualisation: Jansen G

### Availability of data and materials

Not applicable.

### Financial support

This study was supported by Cancer Center Amsterdam grants 07/36 and 2012-1-08.

### Conflicts of interest

A preliminary account of this work was presented at the 2018 Annual Meeting of the American Society for Hematology (J. Cloos *et al*. Blood, vol 132, Supplement 1, Nov 2018, p 3945, abstract).

### Ethical approval and consent to participate

This study only used cell lines, and thus ethical approval and consent to participate do not apply.

### Consent for publication

Not applicable.

### Copyright

© The Author(s) 2023.

### Supplementary Materials

Supplementary Materials
