# Supplementary material for: Statins markedly potentiate aminopeptidase inhibitor activity against (drug-resistant) human acute myeloid leukemia cells
Source: Cancer Drug Resist. 2023 Jul 4;6(3):430–46. doi: 10.20517/cdr.2023.20 (PMC10571057; doi:10.20517/cdr.2023.20)
Supplement: Supplementary file 1 [file cdr-6-3-430-SupplementaryMaterials.pdf]

## Supplementary Materials

**Suppl Figure 1**

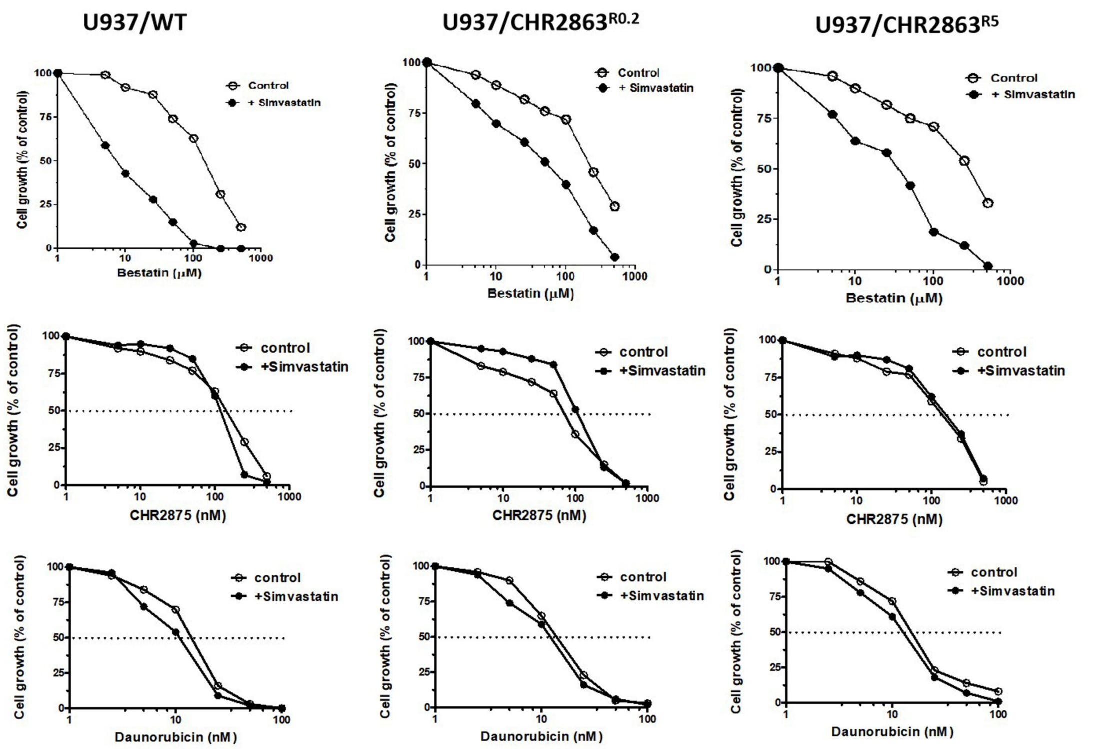


**Supplementary Figure 1.** Growth inhibitory effects of Bestatin (top row), CHR2875 (middle row) and Daunorubicin (bottom row) for U937/WT cells (left lane), U937/CHR2863$^{R0.2}$ cells (middle lane) and U937/CHR2863$^{R5}$ cells (right lane) in the absence and presence of maximal in vitro non-toxic concentrations of simvastatin (2 μM, 2.5 μM and 2.5 μM, respectively). Cell growth inhibition was determined after 72 h of drug exposure. Results depicted are the mean of two separate experiments (for Bestatin) and four experiments for CHR2875 and Daunorubicin.

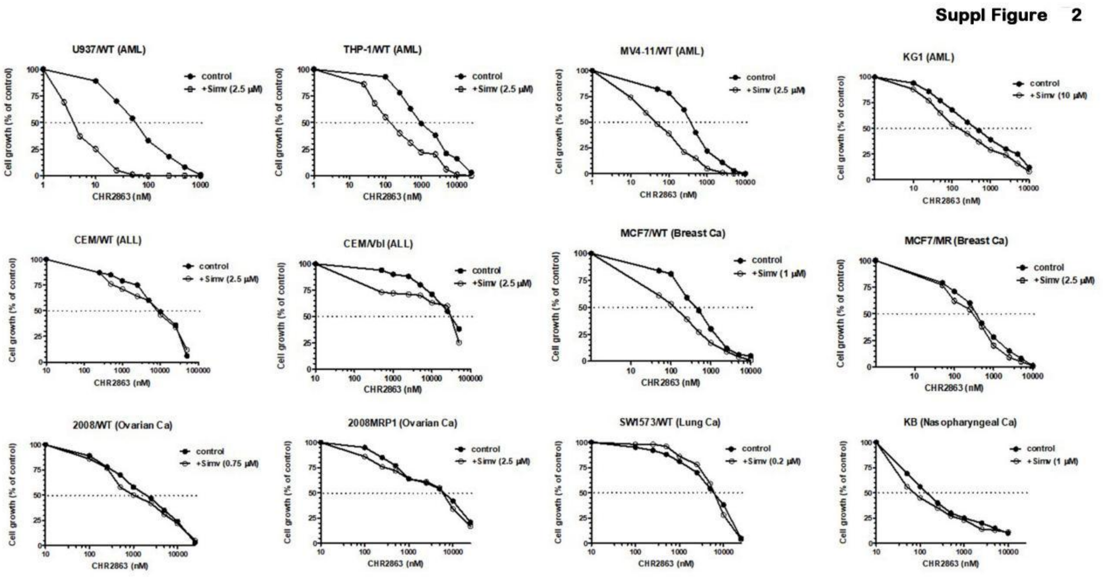


**Supplementary Figure 2.** Growth inhibition curves for selected AML cell lines (U937, THP1, MV4-11 and KG1), ALL cell lines (CEM, CEM/Vbl) and solid tumor cell lines (MCF7/WT, MCF7/MR, 2008, 2008/MRP1, SW1573 and KB) in the absence and presence of maximum in vitro non-toxic concentration of simvastatin (indicated for each cell line). Cell growth inhibition was determined after 72 h of drug exposure. Results depicted are the mean of 3-4 separate experiments.

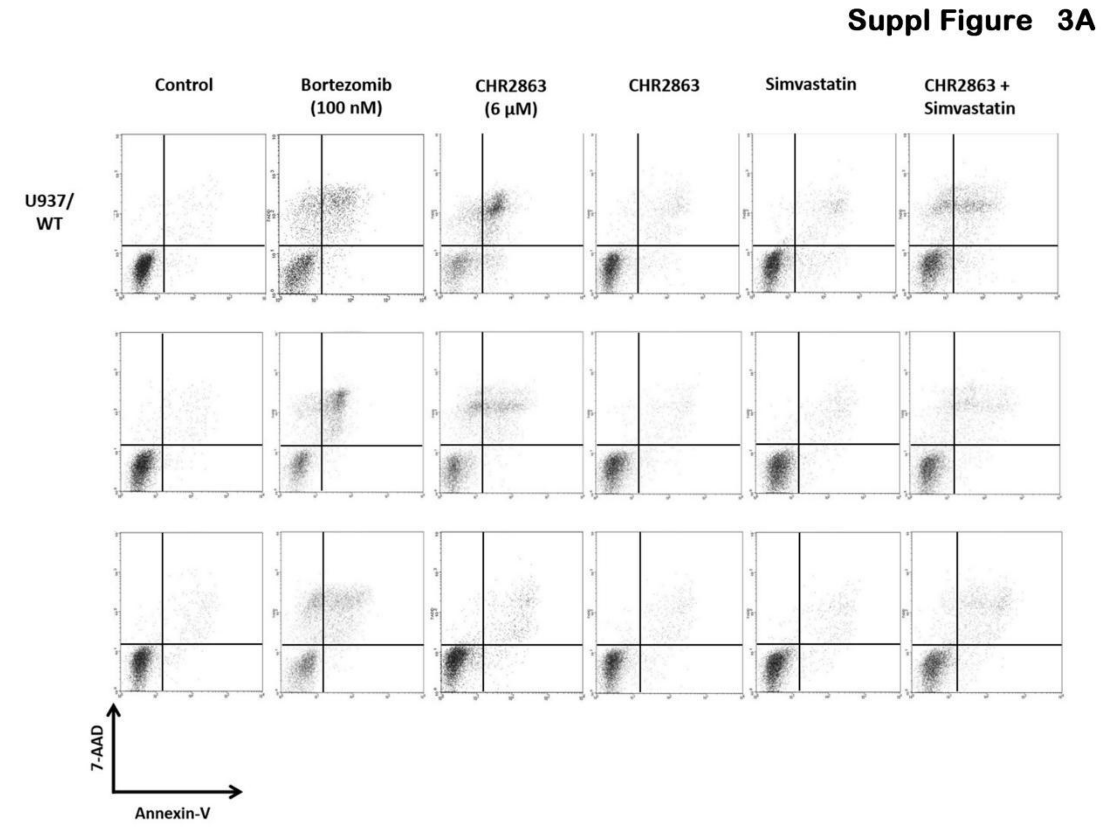


**Supplementary Figure 3A.** Representative FACS analysis plots of apoptosis induction in U937/WT, U937/CHR2863$^{R0.2}$ and U937/CHR2863$^{R5}$ cells following 48 h of exposure to simvastatin and CHR2863 combinations (as described in Figure 5) and then analyzed for Annexin-V/7AAD staining. Cells treated for 24 h with 0.1 µM bortezomib served as apoptosis induction control. Lower and upper right quadrant represents early apoptotic and late apoptotic cells, respectively.

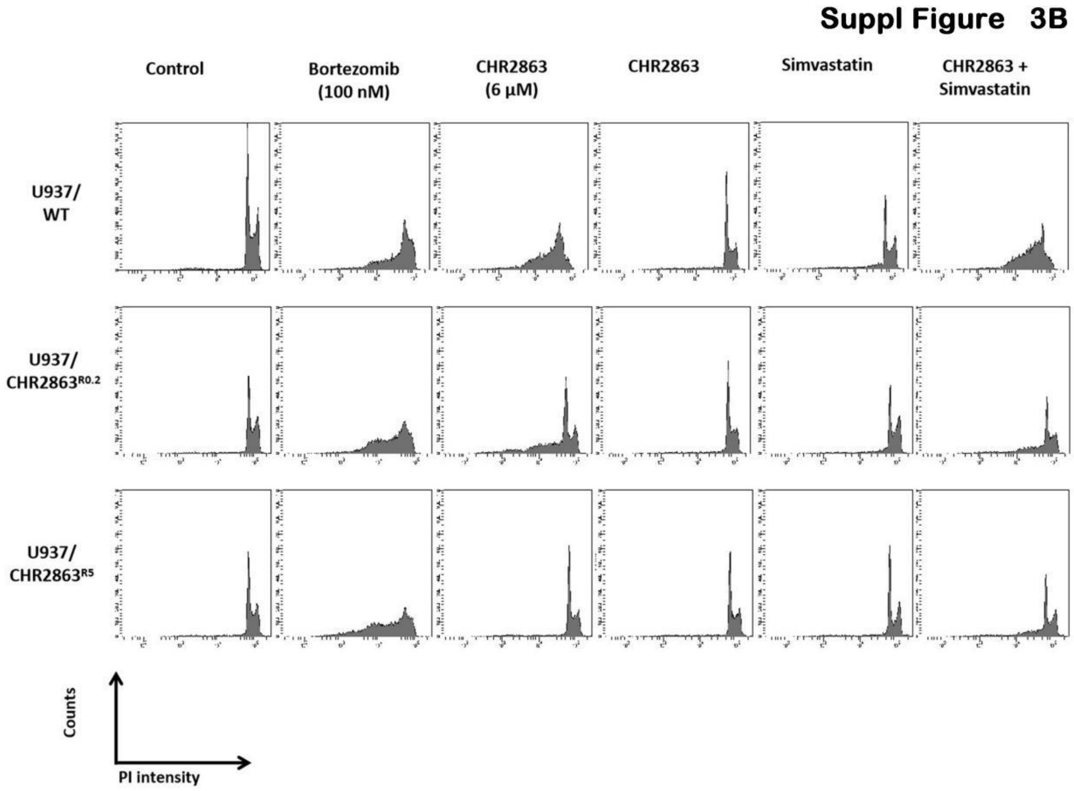


**Supplementary Figure 3B.** Representative alterations in cell cycle distribution of U937/WT, U937/CHR2863$^{R0.2}$ and U937/CHR2863$^{R5}$ cells following 48 h of exposure to simvastatin and CHR2863 combinations (as described in Figure 5) and then analyzed for PI staining. Cells treated for 24 hr with 0.1 µM bortezomib served as control.

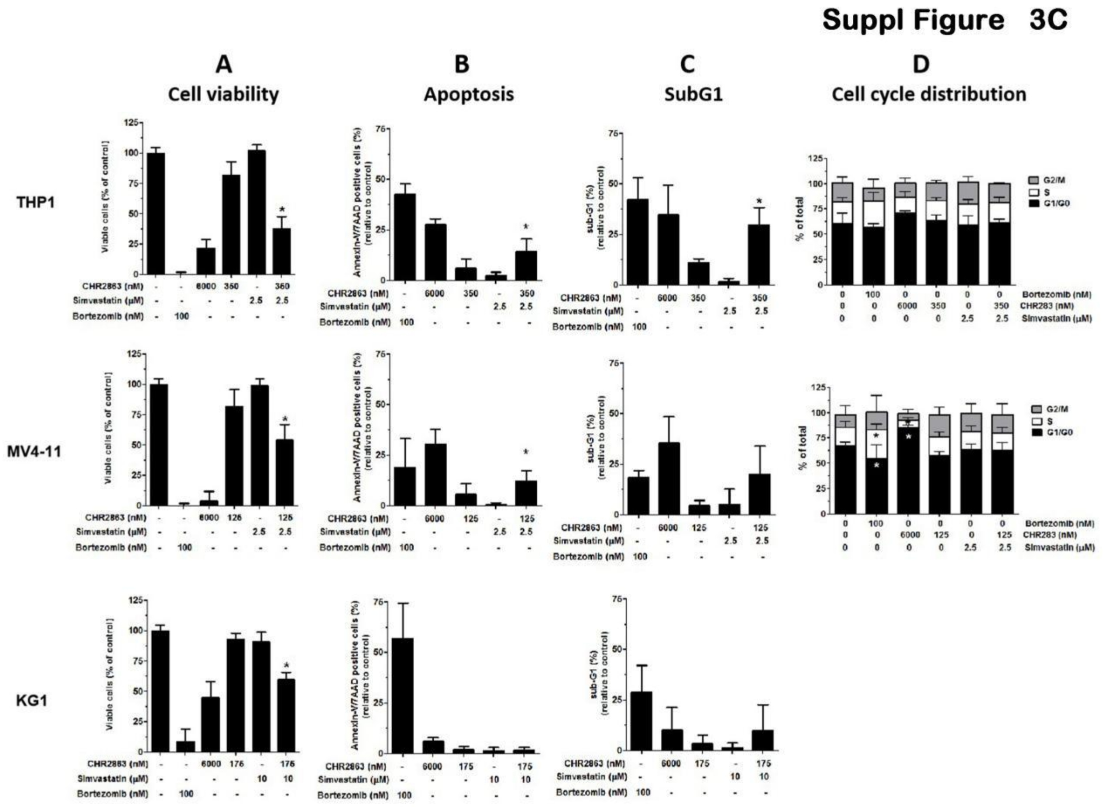


**Supplementary Figure 3C.** Effect of simvastatin and CHR2863 combinations on cell viability, apoptosis induction and cell cycle distribution in AML cell lines THP1, MV4-11 and KG1 cells. Cells were incubated for 48 h with the indicated concentrations simvastatin, CHR2863 and their combination and assessed for their impact on (A) cell viability, (B) apoptosis induction, (C) sub-G1 fraction and (D) cell cycle distribution. Cells incubated for 24 h with bortezomib or 48 h with 6 µM CHR2863 served as the control for cell growth inhibition and apoptosis induction. Percentages of apoptotic cells in control untreated THP1, MV4-11 and KG1 cells were: 7.2% ± 1.1%, 9.8% ± 3.8% and 7.9% ± 3.7%, respectively. Sub-G1 fraction in control untreated THP1, MV4-11 and KG1 cells was 9.8% ± 3.8%, 10.4% ± 2.1% and 11.4% ± 5.2%, respectively. Results depicted are the mean ± SD of 4-5 independent experiments. *Combination statistically significant ($P < 0.05$) different compared to single drugs.

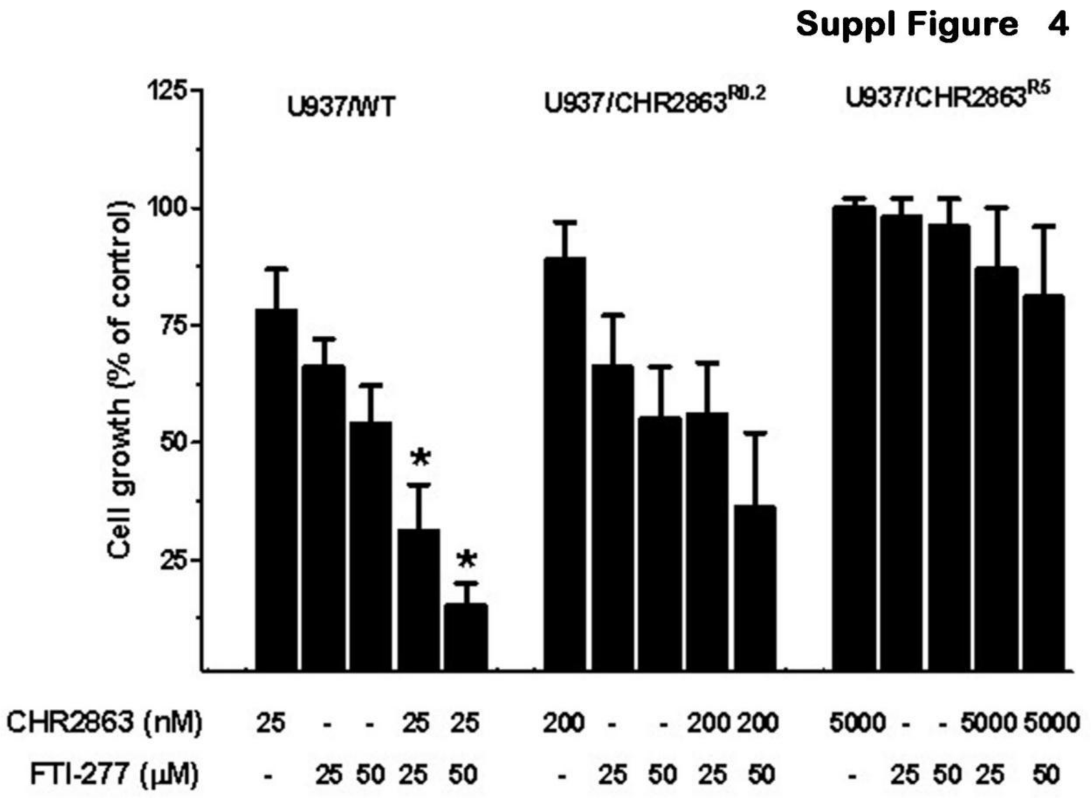


**Supplementary Figure 4.** Effect of farnesyltransferase inhibitor FTI-277 on CHR2863 activity. U937/WT, U937/CHR2863$^{R0.2}$ and U937/CHR2863$^{R5}$ cells were incubated for 72 h with the indicated concentrations FTI-277, CHR2863 and their combination and assessed for the growth inhibitory effects. Results depicted are the mean ± SD of 4 separate experiments. *combination statistically significant ($P < 0.05$) different compared to single drugs.

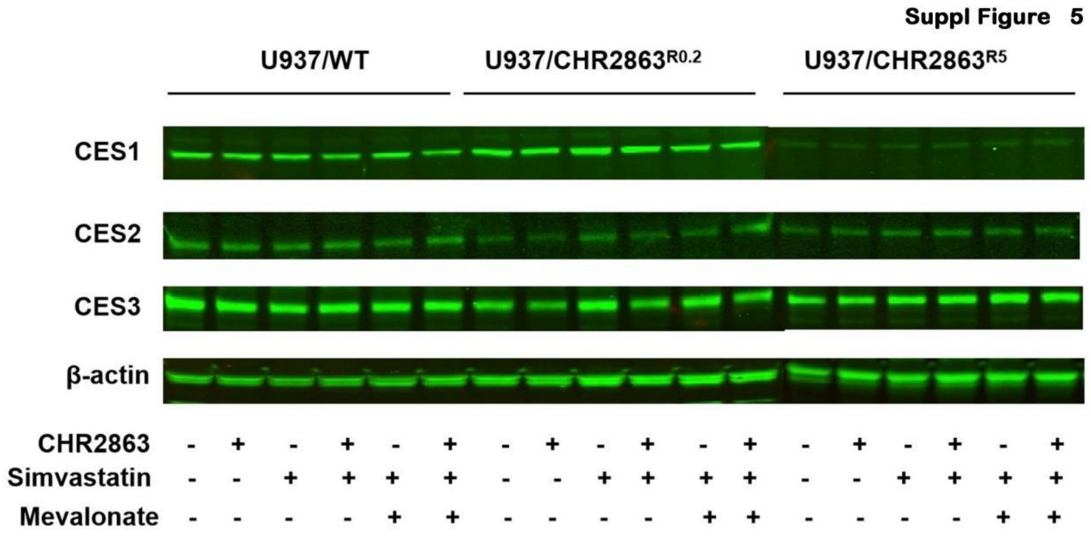

**Supplementary Figure 5.** Effect of CHR2863, simvastatin and their combination on carboxyl esterase-1, -2 and 3 expression. Under conditions described in Figure 5, U937/WT, U937/CHR2863$^{R0.2}$ and U937/CHR2863$^{R5}$ cells (3 × $10^5$/ml in 10 mL medium) were incubated for 48 h with non-toxic concentrations simvastatin (2 µM, 2.5 µM and 2.5 µM, respectively), CHR2863 (25 nM, 250 nM and 5 µM, respectively) and 100 µM mevalonic acid (and in combination). Cell lysates were prepared and analyzed for carboxyl esterase (CES) 1, CES2 and CES3 expression. β-Actin expression served as loading control.

Suppl Figure 6

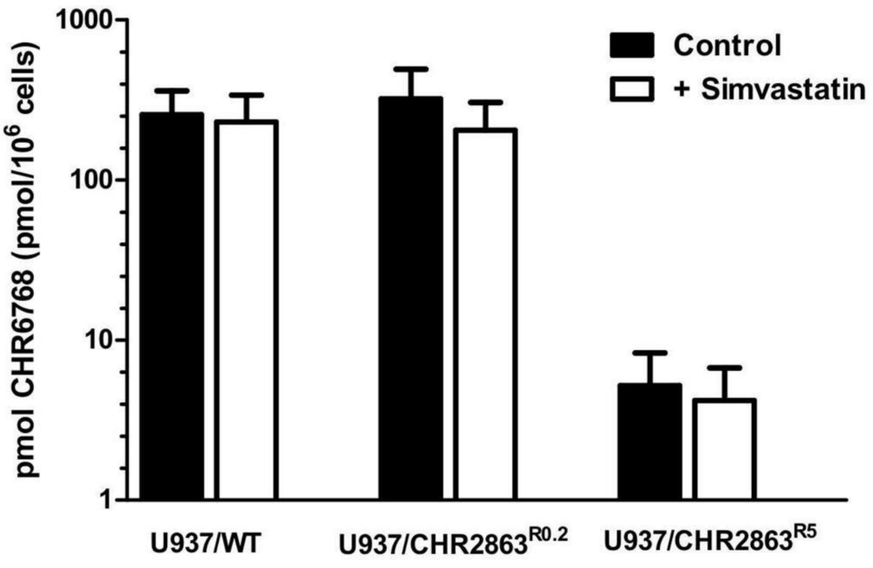


**Supplementary Figure 6.** Effect of simvastatin on the conversion of CHR2863 to its active metabolite CHR6768. U937/WT, U937/CHR2863$^{R0.2}$ and U937/CHR2863$^{R5}$ cells (3 × $10^5$/ml in 10 mL medium) were preincubated for 1 hour with simvastatin (2 µM, 2.5 µM and 2.5 µM, respectively) followed by incubation with 6 µM CHR2863 for 6 h at 37 °C. Then, cells were harvested and processed for LC/MS-MS analysis of CHR6768 metabolite formation. Results depicted are the mean ± SD of 6-8 separate experiments.

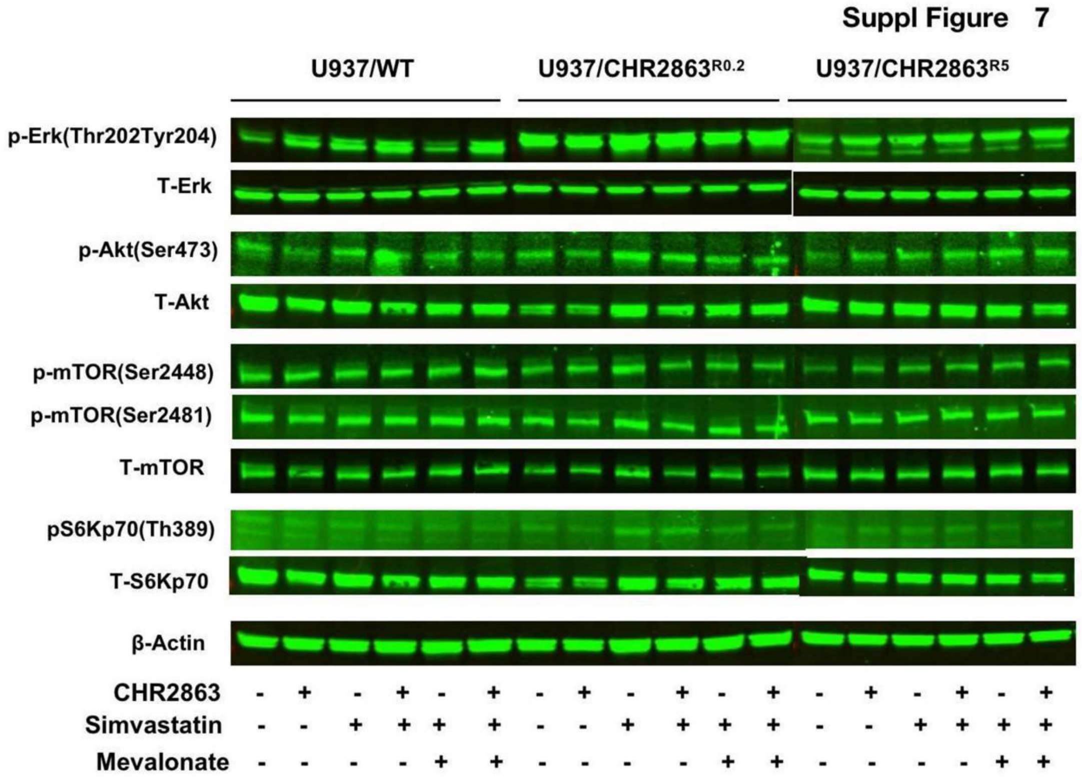


**Supplementary Figure 7.** Effect of simvastatin, CHR2863, mevalonic acid, and their combination on the levels of total and phosphorylated Erk, Akt mTOR and S6K. Under conditions described in Figure 5, U937/WT, U937/CHR2863$^{R0.2}$ and U937/CHR2863$^{R5}$ cells (3 × $10^5$/ml in 10 mL medium) were incubated for 48 h with non-toxic concentrations of simvastatin (2 µM, 2.5 µM and 2.5 µM, respectively), CHR2863 (25 nM, 250 nM and 5 µM, respectively) and 100 µM mevalonic acid as well as their combination. Cell lysates were prepared and analyzed for the levels of total and phosphorylated Erk, Akt, mTOR and S6K. β-actin expression served as the loading control.
